# Dr. Philippe Ricord

**Published:** 1889-10

**Authors:** 


### Obituary.

DR. PHILIPPE RICORD, who died on Wednesday, Oct. 23, was born on December 10, 1800, at Baltimore, Maryland. His was a French family which settled in the United States and has prospered here, the name being well represented in the middle Atlantic States. Some years since a Ricord was the Mayor of Newark, N. J. Philippe, at the age of 20, was sent to Paris to study law, but intead became enthusiastic for surgery after hearing one of Dupuytren's lectures. In 1823, having become an interne, he was assigned to the celebrated surgeon's hospital, but soon lost favour owing to a pun which has remained legendary in the Latin quarter. Dupuytren, one day at his morning clinic, said of a patient dead during night, "Il a du mourir du delirium tremens (tres-mince)." "Pas si mince" (not so thin), whispered young Philippe, "puisqu'il en est mort." The head surgeon overheard the remark and severely rebuked the punster, adding that with such disposition Arnal's performance at the Varietes would suit him better than a surgical clinic. Ricord at once left Dupuytren's services, and it is said the two never became reconciled. In 1826 he graduated as a doctor of medicine with a thesis on "Sundry Propositions on Surgery," offering no particular interest. But just as he was going to push his way on to Paris, the loss of his father's fortune compelled him to settle in a small country town near Orleans. He left it, however, on the first occasion, he having in 1826 secured by competition the post of surgeon in the Pitie hospital. Finally, in 1831, he was appointed head surgeon to the Midi Hospital (male syphilitics), where he remained until 1860, when the age limit obliged him to retire. His contributions to medical sciences have been numerous and most important, but it is chiefly as a syphilograph that he became celebrated. He was the first to show that gonorrhœa and syphilis are distinct affections, and to clearly establish the evolution and different stages of the syphilitic infection. His principal works are on the bivalvular speculum (1833), blennorrhagia in woman (1834), monography of the chancre (1837), treatise on venereal diseases (1838-1863), blennorrhagic ophthalmia (1842), syphilisation and contagiousness of secondary stages (1853). Besides he sent many memoirs to the Academy of Medicine and contributions to the medical press. He also wrote considerable annotations for the French translation of Hunter's treatise on venereal diseases. At one time he had with English physicians quite a controversy, which occasioned his visit to England, when—the French claim—he converted his opponents to his views. Strange enough, notwithstanding his world-wide reputation, it was only in 1850 he was elected to the Academy of Medicine, and he never was given a chair at the Faculty. It was at his hospital he did his teaching before immense classes of voluntary hearers, who loved him as much for his kindness as for his wit and quaint sayings. He was decorated with all the orders of Europe and elsewhere, and scandal has it that the bestowers had personal reasons to be thankful. Ricord would only smile and say he was something like that pedicure who had eradicated corns from many crowned heads in Europe. His last appearance in public was in 1870 during the siege, when at the age of 70 he had charge of the ambulances, and behaved with such gallantry that President Thiers in 1871 made him a grand officer of the Legion of Honor.

Among the last acts of Ricord's life, says the "British Medical Journal," was the ascent of the Eiffel Tower, and his death was hastened by his coming from Versailles to Paris to vote for the Republic in very inclement weather. Having to wait on the platform, he contracted a severe cold, and was attacked with double pneumonia. Only a few days before his death, one of his friends, on seeing him, said to him: "Mais, mon maitre, vous avez bonne mine." His characteristic answer was: "Mon ami, je ne vous conseille pas de pendre des actions dans cette mine-la." A charming "calembour," which showed that to the last he retained all the brightness of his inimitable wit and gayety of spirit. His kindness to young men, his generosity to the poor, his hearty welcome and unbounded liberality to foreigners, and his cordial hospitality to all English visitors did much during all his life to render him and his profession popular with all visitors.

